# Natural Products as Drug Candidates for Redox-Related Human Disease

**DOI:** 10.3390/ph16091294

**Published:** 2023-09-13

**Authors:** Jong H. Kim, Kirkwood M. Land, Canhua Huang, Yuan-Yuan Zhang

**Affiliations:** 1 Foodborne Toxin Detection and Prevention Research Unit, Western Regional Research Center, Agricultural Research Service, United States Department of Agriculture, 800 Buchanan St., Albany, CA 94710, USA; 2 Department of Biological Sciences, University of the Pacific, 3601 Pacific Avenue, Stockton, CA 95211, USA; 3 School of Basic Medical Sciences, Chengdu University of Traditional Chinese Medicine, Chengdu 611137, China; 4 West China School of Basic Medical Sciences and Forensic Medicine, State Key Laboratory of Biotherapy and Cancer Center, West China Hospital and Sichuan University and Collaborative Innovation Center for Biotherapy, Chengdu 610041, China; 5 West China School of Pharmacy, Sichuan University, Chengdu 610041, China; 6 Department of Endocrinology, Affiliated Hospital of Southwest Medical University, Luzhou 646000, China

This Special Issue presented recent progress on natural products that serve as drug candidates for redox-related human diseases. The disruption of cellular redox homeostasis, viz., oxidative stress, results in the development of various human diseases/pathologies. For example, interference of the normal mitochondrial function/respiration generates reactive oxygen species (ROS) in the cell, which are responsible for illnesses such as cardiovascular/neurodegenerative diseases, metabolic disorder or aging [1]. Oxidative stress-associated human diseases further include Parkinson’s disease, Huntington’s disease, inflammation (arthritis), ischemia–reperfusion injury, atherosclerosis, amyotrophic lateral sclerosis, stroke, red blood cell disorder, cancer and cataract, among others. Therefore, the precise regulation of cellular redox homeostasis is crucial for preventing the development of human diseases. Noteworthy, since the identification of the important physiological roles exerted by ROS [2], recent research for redox-related human diseases focuses more on redox signaling and regulation in the cell [3].

Conversely, the disruption of cellular redox homeostasis has been indicated as an effective strategy for the control of infectious pathogens, such as the protozoan parasite *Trichomonas vaginalis* [4]. Due to the microaerophilic lifestyle, *T. vaginalis* and other protozoa are highly susceptible to ROS; thioredoxin-dependent peroxidases play key roles in pathogen defense against oxidative stress. Of note, a recent study showed that phendione (1,10-phenanthroline-5,6-dione) and its silver and copper complexes triggered cellular oxidative damage (thus, redox homeostasis imbalance) in the pathogen, leading to apoptotic-like cell death in *T. vaginalis* [5].

Meanwhile, the invasive fungal pathogen *Aspergillus terreus*, a causative agent for human aspergillosis, possesses intrinsically higher activity of the antioxidant enzyme catalase compared to other fungal pathogens. The robust antioxidant system of *A. terreus* contributes to the resistance of this fungal pathogen to the oxidative stress drug amphotericin B. Therefore, disruption of the antioxidant systems of pathogens could serve as an effective strategy for the control of certain fungal pathogens including *A. terreus*. Enhancement of a pathogen’s oxidative stress using prooxidant compounds, administered alone or in combination with other antifungal drugs, has been proposed as a new therapeutic strategy [6].

Natural products could serve as a potential source of drug candidates for redox-related human diseases, either in their intact form or as templates/leads for more effective structural analogs. For instance, phytochemical compounds, such as polyphenols, terpenoids or glucosinolates, possess therapeutic potential, for which the regulation of glutathione-related cellular processes is one contributing mechanism of action [7]. Redox-active natural products (e.g., sulfur-containing compounds, phenolics, benzo analogs, etc.) can also serve as potent redox cyclers/prooxidants against pathogens, hence inhibiting pathogen growth via the destabilization of pathogens’ antioxidant systems [8].

In this Special Issue, ten works (five original research articles and five reviews) were published on the recent advances in natural products as drug candidates for redox-related human disease.

Wan et al. [9] investigated the repurposing of ciclopirox olamine (CPX), a synthetic antifungal agent for treating tinea corporis, tinea pedis, lichen planus and *Candida albicans*, to cure hepatocellular carcinoma (HCC). In the study, the researchers identified that CPX (1) inhibited the proliferation of HCC cells by arresting the cell cycle, (2) triggered cellular ROS accumulation as well as the downregulation of DJ-1, an oxidative stress sensor responsible for the onset of oxidative stress-related diseases, for example, cancer, neurodegenerative disorders and type 2 diabetes, (3) promoted complete autophagic flux, and (4) induced glycogen clustering in HCC cells. The study provided a new insight into the molecular mechanisms of CPX as an anti-cancer therapeutic agent and a future strategy for treating HCC.

Colon cancer is a common digestive tract malignancy, exhibiting the second highest mortality rate among all tumors [10]. Annexin A1 (ANXA1) is a calcium-regulated membrane-binding protein, which functions as an inhibitor of phospholipase A2, thus regulating inflammation [11]. ANXA1 also plays an important role in the processes of tumorigenesis (growth, invasion, migration and drug resistance) [10]. Wang et al. determined that ANXA1 is responsible for promoting the proliferation of colon cancer cells by regulating the cell cycle. Also, while “honokiol” (an active ingredient of the traditional Chinese herb *Houpo*) caused the autophagic death in colon cancer cells through the stabilization of mitochondrial ROS, ANXA1 antagonized the antitumor activity. Therefore, it is concluded that targeting ANXA1 would enhance the antitumor effect of honokiol by effectively modulating the autophagic signaling pathway [10].

Nanoparticle-based drug delivery systems were developed by Chen et al. [12] whereby gold nanoparticles containing resveratrol (natural phenol) (RGNPs) were designed as an anti-aging agent to delay cataracts, a serious eye disease. The designed RGNPs had the capacity to inhibit oxidative stress damage triggered by hydrogen peroxide, ROS, etc. Noteworthy, RGNPs delayed oxidative stress-induced cellular senescence by decreasing the levels of p16 and p21 proteins (senescence markers) and reducing the ratio of BAX/BCL-2 (pro-apoptotic family) as well as the senescence-associated secretory phenotype. It was determined that RGNPs reduced cell senescence and delayed cataracts through activating the Sirt1 (silent information regulator transcript-1)/Nrf2 (nuclear erythroid factor 2-related factor 2) signaling pathway [12].

In that aspect, Niu et al. [13] provided a review on the therapeutic role of *Lycium barbarum* L. polysaccharides (LBPs) in oxidative stress-related ocular diseases. LBPs, which possess antioxidant activity, were extracted from *L. barbarum*. While LBPs exerted various therapeutic effects such as immunomodulatory-, neuroprotective-, antitumor-, anti-inflammatory effects, etc., they also provided therapeutic potential towards ocular diseases, for example, diabetic retinopathy, hypertensive neuroretinopathy, age-related macular degeneration, retinitis pigmentosa, retinal ischemia/reperfusion injury, glaucoma, dry eye syndrome and diabetic cataracts [13].

By using *Jaeumgeonbi-Tang* (JGT), a traditional herbal medicine, Kim et al. [14] examined the pharmacological properties of JGT in chronic subjective dizziness (CSD) patients. They analyzed the levels of oxidative stressors, antioxidants, and stress hormones in the serum, where the levels of lipid peroxidation (but not nitric oxide) were significantly lowered in the JGT-receiving group. JGT prevented the decline of serum total glutathione contents and antioxidant capacity as well as increasing the activities of superoxide dismutase and catalase, crucial antioxidant enzymes in the cell [14]. Furthermore, the levels of stress hormones in serum such as cortisol, adrenaline, and serotonin were notably normalized by JGT, while noradrenaline levels were not affected by the treatment [14].

The monoterpene alcohol (–)-isopulegol ((1*R*,3*R*,4*S*)-*p*-menth-8-en-3-ol) could be transformed to derivatives possessing antiviral, analgesic, antioxidant, antimicrobial and antiproliferative activities. Ivshina et al. [15] established the biotransformation measures of the monoterpenoid (–)-isopulegol using actinobacteria of the genus *Rhodococcus*, whereby the production of 10-hydroxy and 10-carboxy derivatives was feasible; these compounds possess antitumor activity and act as respiratory stimulants [15].

Mitochondrial inefficiency/dysfunction (e.g., mutations in mitochondrial DNA, excessive ROS accumulation, etc.) have been implicated in neurodegenerative diseases, such as Alzheimer’s disease, Parkinson’s disease and Huntington’s disease, among others [16]. Therefore, targeting mitochondria could provide a novel therapeutic opportunity for curing neurodegenerative diseases. Zhang et al. [16] reviewed on the recent progress in mitochondrial neurotherapeutics via the application of plant-derived natural products. The mechanisms of action of plant-derived natural products on mitochondria was also discussed, where natural products, such as ginger extract (6-gingerol, 6-chrysophanol), saffron, asiaticoside extract, carnosic acid, flavonoids (quinic acid, resveratrol), polyphenols (morin, mangiferin (4-Glucosyl-1,3,6,7-tetrahydroxyxanthone)), ginsenoside Rg1 (a tetracyclic triterpenoid derivative), black tea extracts, etc., stimulated mitochondrial biogenesis, regulated mitochondrial fusion and fission, improved mitochondrial bioenergetics, prevented mitochondrial oxidative stress, modulated mitochondrial calcium (Ca^2+^) homeostasis, sustained mitochondrial membrane potential (∆ψm) and also maintained mitochondrial DNA stability. Currently, the precise mechanisms of therapeutic action of plant-derived natural products require future in-depth investigation.

Jia et al. [17] provided a review on the nano-based drug delivery of polyphenolic compounds for cancer treatment. Although natural polyphenols or their structural derivates are potent antioxidants and therefore, have been shown to exert cancer-preventative effects, the low stability, weak targeting ability, poor solubility, and low bioavailability of the molecules limits their therapeutic efficacy [17]. The review discussed the advantages of applying nanomaterials, such as (1) an increase in the aqueous solubility, and (2) an enhancement in the targeting ability of polyphenolic compounds. The review featured the nanocarrier-mediated delivery of natural polyphenolic compounds in cancer therapy, including liposome-mediated delivery of polyphenols and micelles/nanogels as nanocarriers for drug delivery.

Studies have shown further that autophagy and ROS regulate each other, which affects the progression of cancer, whereas natural phytochemicals that can modulate ROS and autophagy possess therapeutic potential [18,19]. Dong et al. [18] provided a review on various natural phytochemicals that target ROS and autophagy for cancer therapy. The types of anti-cancer phytochemicals featured in the review included celastrol (tripterine; quinine methide triterpenoid), curcumin, allicin, erianin (low-molecular-weight bibenzyl natural product), chrysin (5,7-di-oh-flavone), isoorientin (a glucoside composed of luteolin), capsaicin, pristimerin (quinone methide triterpenoid), neohesperidin (flavanone glycoside), polyphyllins, magnoflorine (quaternary aporphine alkaloid), baicalin, bigelovin (sesquiterpene lactone), diosgenin, trichosanthin, piperlongumine, betulinic acid (pentacyclic triterpenoid), and Rg3-enriched red ginseng extract (Rg3-RGE). As discussed previously, encapsulation of the phytochemicals in nanoparticles could modulate the speed of drug release and improve the drug bioavailability.

Finally, Li et al. [20] reviewed therapeutic polyphenols including their regulatory mechanisms, which are linked to the modulation of cellular redox homeostasis/antitumor characteristics. The types of polyphenols mediating antioxidant effects in cancer therapy included kaempferol, resveratrol and catechins, among others. Of note, therapeutic polyphenols, such as curcumin, wogonin and resveratrol, were suppressive towards cancer via the promotion of oxidative stress in the cancer cells [20]. Structural modifications, e.g., esterification, methylation and glycosylation, were proposed to prevent the degradation as well as the enhancement of the bioactivities of polyphenols. As with other studies, nano strategies were also suggested to overcome the low water solubility, poor stability and nontargeting ability of polyphenols.

In summary, the research articles and reviews presented in this Special Issue provide useful information/insight and illuminate recent progress on natural products that serve as drug candidates for redox-related human diseases. Identification of new, safe molecules and cellular targets, as well as elucidation of their precise mechanisms of action, will further the effective control of redox-related human diseases.

## References

[1] Zuo J., Zhang Z., Luo M., Zhou L., Nice E.C., Zhang W., Wang C., Huang C. (2022). Redox signaling at the crossroads of human health and disease. MedComm..

[2] Khan A.U., Wilson T. (1995). Reactive oxygen species as cellular messengers. Chem. Biol..

[3] Kuczyńska M., Jakubek P., Bartoszek A. (2022). More than just antioxidants: Redox-active components and mechanisms shaping redox signalling network. Antioxidants.

[4] Leitsch D., Williams C.F., Hrdý I. (2018). Redox pathways as drug targets in microaerophilic parasites. Trends Parasitol..

[5] Vargas Rigo G., Willig J.B., Devereux M., McCann M., Souza dos Santos A.L., Tasca T. (2023). Oxidative damage by 1,10-phenanthroline-5,6-dione and its silver and copper complexes lead to apoptotic-like death in *Trichomonas vaginalis*. Res. Microbiol..

[6] Vallières C., Golinelli-Cohen M.-P., Guittet O., Lepoivre M., Huang M.-E., Vernis L. (2023). Redox-based strategies against infections by eukaryotic pathogens. Genes.

[7] Di Giacomo C., Malfa G.A., Tomasello B., Bianchi S., Acquaviva R. (2023). Natural compounds and glutathione: Beyond mere antioxidants. Antioxidants.

[8] Kim J.H., Chan K., Faria N.C.G., Martins M.d.L., Campbell B. (2012). Targeting the oxidative stress response system of fungi with redox-potent chemosensitizing agents. Front. Microbiol..

[9] Wan X., Xiang J., Fan H., Jiang Y., Lu Y., Zhang C., Zhang Y., Chen Q., Lei Y. (2023). Ciclopirox olamine induces proliferation inhibition and protective autophagy in hepatocellular carcinoma. Pharmaceuticals.

[10] Wang X., Shao G., Hong X., Shi Y., Zheng Y., Yu Y., Fu C. (2023). Targeting annexin A1 as a druggable player to enhance the anti-tumor role of honokiol in colon cancer through autophagic pathway. Pharmaceuticals.

[11] Shao G., Zhou H., Zhang Q., Jin Y., Fu C. (2019). Advancements of annexin A1 in inflammation and tumorigenesis. OncoTargets Ther..

[12] Chen Q., Gu P., Liu X., Hu S., Zheng H., Liu T., Li C. (2023). Gold nanoparticles encapsulated resveratrol as an anti-aging agent to delay cataract development. Pharmaceuticals.

[13] Niu Y., Zhang G., Sun X., He S., Dou G. (2023). Distinct role of *Lycium barbarum* L. polysaccharides in oxidative stress-related ocular diseases. Pharmaceuticals.

[14] Kim C.-y., Kim H.-G., Lee H.W., Seol I.-C., Kim Y.-S., Choi H.I., Park M.S., Yoo H.-R. (2022). Pharmacological properties of *Jaeumgeonbi-Tang* on redox system and stress-related hormones in chronic subjective dizziness: A randomized, double-blind, parallel-group, placebo-controlled trial. Pharmaceuticals.

[15] Ivshina I.B., Luchnikova N.A., Maltseva P.Y., Ilyina I.V., Volcho K.P., Gatilov Y.V., Korchagina D.V., Kostrikina N.A., Sorokin V.V., Mulyukin A.L. (2022). Biotransformation of (–)-isopulegol by *Rhodococcus rhodochrous*. Pharmaceuticals.

[16] Zhang X., Wang L., Li B., Shi J., Xu J., Yuan M. (2023). Targeting mitochondrial dysfunction in neurodegenerative diseases: Expanding the therapeutic approaches by plant-derived natural products. Pharmaceuticals.

[17] Jia W., Zhou L., Li L., Zhou P., Shen Z. (2023). Nano-based drug delivery of polyphenolic compounds for cancer treatment: Progress, opportunities, and challenges. Pharmaceuticals.

[18] Dong L., He J., Luo L., Wang K. (2023). Targeting the interplay of autophagy and ROS for cancer therapy: An updated overview on phytochemicals. Pharmaceuticals.

[19] Klionsky D.J., Petroni G., Amaravadi R.K., Baehrecke E.H., Ballabio A., Boya P., Bravo-San Pedro J.M., Cadwell K., Cecconi F., Choi A.M.K. (2021). Autophagy in major human diseases. EMBO J..

[20] Li L., Jin P., Guan Y., Luo M., Wang Y., He B., Li B., He K., Cao J., Huang C. (2022). Exploiting polyphenol-mediated redox reorientation in cancer therapy. Pharmaceuticals.

